# Trajectories and Determinants of Physical Activity during COVID-19 Pandemic: A Population-Based Study of Middle-Aged and Elderly Individuals in The Netherlands

**DOI:** 10.3390/nu13113832

**Published:** 2021-10-27

**Authors:** Amy Hofman, Marlou A. M. Limpens, Tosca O. E. de Crom, Mohammad Arfan Ikram, Annemarie I. Luik, Trudy Voortman

**Affiliations:** 1 Department of Epidemiology, Erasmus MC University Medical Center, 3000 CA Rotterdam, The Netherlands; amy.hofman@erasmusmc.nl (A.H.); m.limpens@erasmusmc.nl (M.A.M.L.); t.decrom@erasmusmc.nl (T.O.E.d.C.); m.a.ikram@erasmusmc.nl (M.A.I.); a.luik@erasmusmc.nl (A.I.L.); 2 Department of Child and Adolescent Psychiatry/Psychology, Erasmus MC University Medical Center, 3000 CA Rotterdam, The Netherlands; 3 Division of Human Nutrition and Health, Wageningen University & Research, 6700 HB Wageningen, The Netherlands

**Keywords:** physical activity, lifestyle, trajectories, latent class trajectory analyses, COVID-19, pandemic, lockdown

## Abstract

Physical inactivity is a major public health problem, and there are concerns this might have increased during the COVID-19 pandemic. We aimed to identify distinct trajectories of physical activity over a 6-week period after the first restrictive measures and to explore determinants of these trajectories in a population-based cohort of middle-aged and elderly in the Netherlands (*n* = 5777). We observed that at least 59% of participants did not meet the World Health Organization recommendations for physical activity. Using latent class trajectory analyses over three time points, we identified five distinct trajectories, including four steady trajectories at different levels (very low, low, medium and high) and one increasing trajectory. Using multinomial logistic regression analyses, we observed that, compared to the ‘steadily high’ trajectory, participants in the ‘steadily very low’ trajectory were more often older, lower educated, reporting poorer physical health, more depressive symptoms, consuming a less healthy diet, smoking, and lower alcohol use, and were less often retired. A similar pattern of determinants was seen for those in the increasing trajectory, albeit with smaller effect sizes. Concluding, we observed low levels of physical activity that generally remained during the pandemic. The determinants we described can help identify groups that require additional preventive interventions.

## 1. Introduction

In March 2020, the World Health Organization (WHO) declared COVID-19 to be a pandemic. To curb the pandemic, several regional and national governments responded by encouraging people to stay at home and keep distance from each other. Due to these restrictive measures many health behaviors may have changed, among which physical activity levels [1].

The health benefits of physical activity are indisputable [2,3]. However, more than half of the European population does not meet the WHO guidelines of at least 150 min of moderate or 75 min of vigorous physical activity per week [4]. Several studies have shown a further decrease in physical activity during the COVID-19 pandemic after the first restrictive measures were implemented [5,6,7]. This is a major public health concern as physical activity may lessen the burden or impact of COVID-19 [8], and potentially also of various physical and mental health consequences of the restrictive measures [9,10]. Encouraging individuals to remain physically active during the pandemic should therefore be a public health priority, calling for the identification of determinants reflecting participants who need specific attention for preventive interventions.

So far, only a few studies have identified changes in physical activity during the pandemic. These studies have found that average physical activity levels remained stable or increased within a few weeks after the start of restrictive measures [5,6]. However, these studies mainly focused on average changes on a population-level, while individuals within a population may show different patterns. Different subgroups of the population, e.g., those with different ages, or different statuses of physical and psychosocial health, may have responded differently to the restrictive measures in their behaviors of physical activity.

Therefore, we determined distinct trajectories of physical activity over a 6-week period after the first restrictive measures were announced and explored determinants of these trajectories in a middle-aged and elderly population in the Netherlands.

## 2. Materials and Methods

### 2.1. Study Design and Participants

This study was embedded within the Rotterdam Study, a large ongoing population-based cohort study among residents of the Ommoord area, a suburb in Rotterdam, the Netherlands. The initial cohort started in 1989 and comprised 7983 participants aged 55 years and older. The cohort was subsequently expanded three times with 3011 participants in 2000 (≥55 years), 3932 participants in 2005 (≥45 years), and 3368 participants in 2016 (≥40 years), resulting in a study population of 18,924 participants [11].

On 8 April 2020, 9008 of the 18,924 participants were still alive and participating in the Rotterdam Study. Among those, 8732 participants were not hospitalized or living in a nursing home and were invited to fill out multiple questionnaires during the COVID-19 pandemic [12].

For the current analyses, we used data from the first three questionnaires. The response rate was 71.5% (N = 6242 out of 8732) for the first questionnaire, 64.6% (N = 5640 out of 8.732) for the second questionnaire and 88.2% (N = 4956 out of 5618) for the third questionnaire. For the current study, we excluded participants with no physical activity data from the first questionnaire (N = 465), leaving 5777 participants for analyses.

### 2.2. Measurements

#### 2.2.1. COVID-19 Questionnaire

The COVID-19 questionnaire was sent out three times between 20 April 2020 and 22 May 2020, with an interval of two weeks. The first two questionnaires were sent on paper to all participants. The third questionnaire was sent both digitally and on paper, only to participants who actively agreed to participate. This questionnaire included questions on the following categories: COVID-19 related symptoms and risk factors, mental health and health care utilization during the pandemic. Questions were based on the COVID-19 questionnaire of the Lifelines COVID-19 cohort, which was designed to compare results among similar projects within Europe and including similar questions as used in studies in Denmark and France at that time [13].

#### 2.2.2. Physical Activity

At each time point, participants answered the question how many minutes they spent in moderate-to-vigorous physical activity (e.g., walking, cycling, running) in total in the previous 14 days. Answer options, in line with the LifeLines COVID-19 cohort questionnaire [13], were “less than 50 min”, “50–100 min”, “100–150 min”, “150–180 min”, and “more than 180 min”.

#### 2.2.3. Determinants

For information on potential determinants, we used data from the first COVID-19 questionnaire (time point 1). We included determinants based on the intrapersonal domain of the socio-ecological model, referring to an individuals’ demographic, biological and psychological characteristics [14,15,16].

1. Demographics

Self-report data were available for sex, age, education, and occupation. Educational level was categorized into primary, lower, intermediate and higher education according to the UNESCO classification. Occupational status was categorized into ‘employed’, ‘not employed’, and ‘retired’ [13,17].

2. Physical Health

Participants were asked how they would self-report their overall health status with answer options ‘excellent’, ‘very good’, ‘good’, ‘fair’, and ‘poor’ [13,17].

3. Psychosocial Health

Depressive symptoms were assessed using a shortened version of the Dutch Center for Epidemiological Studies Depression (CES-D) scale [18,19]. The sum score, based on 10 items of the questionnaire, ranges from 0 to 30, with higher scores indicating more depressive symptoms. To assess symptoms of anxiety, the subscale for anxiety of the Hospital Anxiety and Depression Scale (HADS-A) was used [20,21]. The subscale consists of seven items resulting in a total score ranging from 0 to 21, a higher score reflects more symptoms of anxiety. Participants were also asked how worried they have been in the past 14 days about the pandemic. Answer options were on a 10-point Likert scale, with higher scores reflecting more worries about the pandemic [13,22,23].

4. Lifestyle

Participants were asked how healthy their eating behaviour was compared to before the pandemic. Answer options were on a 5-point Likert scale: “much less healthy”, “less healthy”, “just as healthy”, “more healthy” and “much more healthy” [13,24]. Smoking was asked by a single question: “Have you smoked in the last 14 days?”, which could be answered with ‘yes’ or ‘no’. Alcohol use was asked by the question: “Have you used alcohol in the last 14 days? If so, how many glasses per day on average?”. This question could be answered by ‘no’ or ‘yes’, with the number of glasses [13].

### 2.3. Statistical Analysis

The study population was characterized using descriptive statistics. Missing information on potential determinants of activity (<3%, except for worries about the pandemic: 4.3%, and occupational status: 9.1%) was imputed using 10 folded multiple imputation using the mice package in R.

To determine distinct trajectories of physical activity, latent class trajectory analyses were performed using the lcmm package in R. Due to the ordinal nature of the physical activity variable, threshold models were used (using the ‘thresholds’ link function) with fixed effects for time. Models from two to seven latent classes were constructed to investigate which of these best fitted our data. Models of five, six or seven classes were almost equal in terms of fit measures (e.g., Bayesian Information Criterion (BIC) and relative entropy). As the solution with six classes showed one class with a small number of participants (N = 167, 2.9%), we continued our analyses with a parsimonious model of five distinct trajectories of physical activity (BIC: 38615.3, relative entropy: 0.71).

Subsequently, we investigated the associations of potential determinants with the five different trajectories of physical activity using multinomial logistic regression. As potential determinants we included: sex, age, educational level, occupational status, self-perceived health, depressive symptoms, anxiety symptoms, worries related to the pandemic, diet, smoking and alcohol use. Class membership (which trajectory the participant belongs to) was used as the outcome variable. First, analyses were performed using sex- and age adjusted models. Secondly, models were adjusted for all other potential determinants in order to study associations of determinants independent of each other. For sensitivity analyses, we repeated the analyses excluding participants who got infected with COVID-19 during the study period.

Data analyses were performed using R version 4.0.2 (The R Foundation for Statistical Computing, Vienna, Austria) using the mice, lcmm and nnet packages.

## 3. Results

Characteristics of the study population are presented in Table 1. Mean age of the population was 69.4 (standard deviation (SD): 11.5) years and 58% was female. Physical activity levels at baseline were generally low: 3434 participants (59.4%) reported less than 150 min of moderate-to-vigorous physical activity in the past 14 days, of which 1323 (22.9%) participants reported even less than 50 min. Only 1673 (29.0%) reported more than 180 min. Descriptive information on physical activity at all three time points is presented in Supplementary Table S1.

### 3.1. Physical Activity Trajectories

**Figure 1:**
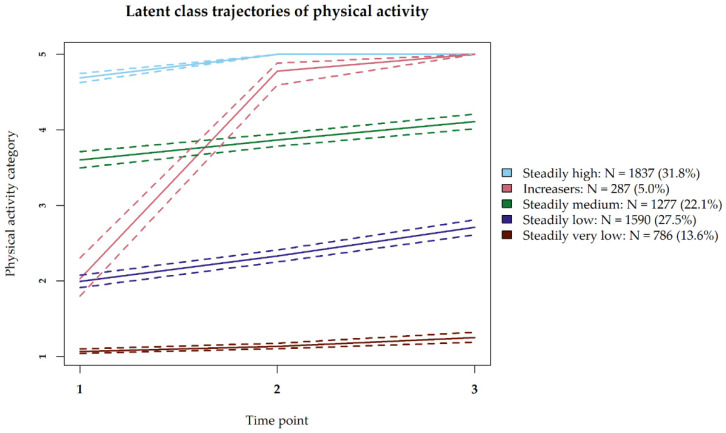
Trajectories of physical activity during COVID-19 pandemic. Question asked at each time point: “During the past 14 days, how many minutes did you exercise (moderately) intensively in total (walking, cycling, running)?”. Physical activity categories refer to the different answer options: 1 ‘Less than 50 min’, 2 ‘50–100 min’, 3 ‘100–150 min’, 4 ‘150–180 min’, 5 ‘More than 180 min’.

Five distinct trajectories were defined (Figure 1, Supplementary Table S2). Four trajectories were steady over time, although a slight increase was shown in each group. A total of 1837 (32%) participants belonged to the trajectory that reported ‘steadily high’ physical activity. Another 1277 (22%) participants reported ‘steadily medium’ levels, and 1590 (28%) participants reported ‘steadily low’ levels. A total of 786 (14%) participants reported ‘steadily very low’ levels of physical activity. Finally, 287 (5%) participants increased their physical activity during the pandemic from the level of the ‘steadily low’ to the ‘steadily high’ group, referred to as ‘increasers.

### 3.2. Determinants of Trajectories

The trajectory of participants who reported ‘steadily high’ physical activity levels over time was used as reference group for all analyses, in order to investigate which determinants were associated with less optimal trajectories. Results of sex- and age adjusted models are presented in Table 2, and of multivariate models in Table 3.

#### 3.2.1. Steadily Medium, Low and Very Low Trajectories

Based on multivariate models, participants in the ‘steadily very low’ trajectory were more often older (odds ratio (OR) = 2.22, 95%-confidence interval 1.94–2.54, per 10 years of age increase), lower educated (e.g., OR = 3.64, 2.45–5.40 for primary versus higher education), and less often retired (OR = 0.38, 0.27–0.53, versus employed) (Table 3). Regarding health status, participants with poorer self-perceived health (e.g., OR = 8.56, 6.08–12.08 for poor/fair health versus very good/excellent health) or more depressive symptoms (OR = 1.13, 1.09–1.17, per point increase on CES-D scale) more often belonged to the ‘steadily very low’ trajectory. In contrast, in multivariable models, those with more anxiety symptoms less often belonged to the ‘steadily very low’ trajectory (OR = 0.90, 0.86–0.94, per point increase on HADS scale). Regarding lifestyle factors, participants who reported to eat less healthy during the pandemic also more often reported ‘steadily very low’ physical activity levels (OR = 2.17, 1.44–3.27, versus those who ate as healthy as before), even as participants who smoked (OR = 2.30, 1.73–3.05, versus non-smokers), whereas participants who used more alcohol were less likely to be in the ‘steadily very low’ trajectory (OR = 0.89, 0.82–0.96, per glass of alcohol per day).

Associations of potential determinants with the ‘steadily medium’ and ‘steadily low’ trajectories showed effect estimates in the same direction. Effect sizes were largest for the ‘steadily very low’, followed by the ‘steadily low’ and ‘steadily medium’ trajectory (Table 3).

In sex- and age adjusted models, associations were largely in the same direction (Table 2). However, without adjustment for the other potential determinants, participants with more anxiety symptoms were more likely instead of less likely to be in a trajectory representing lower levels of physical activity (e.g., for the ‘steadily very low’ trajectory, OR = 1.09, 1.06–1.12, per point on HADS scale). The change in direction in multivariate models was mainly explained by mutual adjustment for depressive symptoms. In sex- and age adjusted models, we also observed that women and unemployed participants were more often in trajectories with lower physical activity levels (Table 2), but these associations were explained by other variables in the multivariate analysis.

#### 3.2.2. Increasers

In multivariate models, we observed that lower educated participants (OR = 1.94, 1.13–1.35 for primary, versus higher education), and participants with poorer health (e.g., OR = 1.96, 1.17–3.30 for poor/fair health, versus very good/excellent health) were more likely to be in the ‘increasers’ trajectory, although effect sizes were smaller as compared to the ‘steadily medium’, ‘steadily low’ and ‘steadily very low’ trajectories (Table 3). Moreover, participants with more depressive symptoms (OR = 1.06, 1.01–1.11, per point increase on CES-D scale), and more worries about the pandemic (OR = 1.07, 1.01–1.14, per point on 10-point Likert scale) more often belonged to the ‘increasers’ trajectory, while participants with more anxiety symptoms (OR = 0.93, 0.87–0.99, per point increase on HADS scale) were less often in the ‘increasers’ trajectory.

Again, associations were largely similar to the sex- and age adjusted models, except for anxiety symptoms (Table 2).

In post hoc analyses we additionally compared the ‘increasers’ trajectory with the ‘steadily low’ trajectory as reference, as these trajectories start at the same baseline physical activity level. In multivariate models, we observed that older participants (OR = 0.62, 0.51–0.75, per 10 years of age increase), lower educated participants (e.g., OR = 0.62, 0.44–0.88 for lower education, versus higher education) and those who reported poor/fair health (OR = 0.50, 0.30–0.84, versus very good/excellent health) were less often in the ‘increasers’ trajectory than in the ‘steadily low’ trajectory (Supplementary Table S3).

### 3.3. Sensitivity Analysis

After exclusion of participants who reported a COVID-19 infection during the study period (n = 92), results did not change (data not shown).

## 4. Discussion

In this large population-based cohort of middle-aged and elderly individuals, the majority of participants reported less than 150 min of moderate and vigorous physical activity in the past 14 days, meaning that they did not meet the WHO physical activity guidelines [25]. Five distinct trajectories of physical activity over a 6-week period during the pandemic were identified. Four trajectories were relatively steady over time, although a slight increase was shown in each group. The fifth group started at a low physical activity level, but increased steeply over time. We identified determinants of these trajectories relating to demographics, physical health, psychosocial health and lifestyle.

Various studies have found a significant drop in physical activity levels during the pandemic compared to before [5,6,26]. We could not directly make this comparison due to lack of information on physical activity levels before the pandemic from the same study sample. Although, according to national data on physical activity levels in the Netherlands in 2019, 49% of the adult population did not meet physical activity guidelines prior to the pandemic, while at least 59.4% of the participants in our study did not meet these guidelines, which indeed suggests a drop in physical activity levels during the pandemic compared to before [27]. In line with our findings, studies that determined physical activity levels after the start of the pandemic found that average physical activity levels either remained stable or increased over time [5,6,28]. The steady levels of physical activity that we observed could be explained by the fact that the restrictive measures in the Netherlands have not changed during our study period. Also, restrictive measures in the Netherlands were relatively mild, i.e., individuals were allowed to go outside for unlimited time to perform physical activity during the pandemic. Previously, Tison et al. have shown that the severity of restrictive measures was of large impact on physical activity in terms of step counts [6]. For example, in Italy, which had a full lockdown, a decrease of 48.7% was measured while in Sweden, which had less strict restrictive measures, a decrease of only 6.9% was observed. As restrictive measures were fixed during our study period, we assume that participants have been able to maintain their physical activity levels during our study period.

The determinants we identified are largely in line with determinants of physical inactivity in general. Furthermore, as most trajectories in our population were stable over time, we presume that identified determinants are representatives of both baseline physical activity and the trajectories. In line with previous findings, we identified demographic and physical health determinants of physical activity trajectories such as older age, lower socioeconomic status and poorer health/disabilities [25,29]. Also, more depressive symptoms were associated with lower physical activity levels in our study, which is in line with previous findings before and during pandemic [30,31,32]. Remarkably, anxiety symptoms were associated with lower trajectories of physical activity in the sex- and age adjusted model, but with higher trajectories in the multivariate model. This change in direction was mainly explained by mutual adjustment for depressive symptoms. We might speculate that anxiety may have been a trigger to be physically active during the pandemic as a healthy lifestyle was suggested to be protective against severe symptoms of COVID-19. Regarding lifestyle factors, those who reported consuming a (much) less healthy diet compared to before the pandemic were more often in trajectories with lower levels of physical activity. This emphasizes the co-occurrence of unhealthy lifestyle factors and the need for combined lifestyle interventions. Also smoking was associated with lower trajectories of physical activity, while alcohol consumption was associated with higher trajectories of physical activity, which is both in line with previous findings [33,34]. The determinants we could assess were all on the individual level. However, from a social-ecological perspective, determinants on other levels, such as interpersonal, organizational and policy factors, may also affect population physical activity levels [14,15,16]. Future studies are warranted to study the additional and interrelating effects of these higher levels factors on individuals’ physical activity during the pandemic.

Determinants of lower trajectories of physical activity during the pandemic largely overlap with those established before the pandemic, emphasizing that these groups are also more vulnerable under these unusual circumstances. Moreover, many of these determinants may also be direct risk factors for chronic diseases in general and specifically to the severity of COVID-19 symptoms. This could lead to enhanced health inequities due to the pandemic. Also, we have shown again that other poor lifestyle factors relate to lower levels of physical activity, which emphasizes the urge for combined interventions to these groups at higher risk of poorer health and lifestyle.

Strengths of this study include the relatively large sample size, repeated measures of physical activity during the pandemic, and the embeddedness within a population-based cohort which might enhance the representativeness of the sample. However, several limitations should be taken into account when interpreting the results. First, physical activity levels could not be directly compared to levels before the pandemic. Second, physical activity was self-reported and measured based on a single question with categorical answer options. Therefore, estimates of physical activity levels may be inaccurate due to e.g., recall bias and categorical answer options may have led to loss of information. Lastly, over the 6-week period that physical activity levels were assessed in this study, restrictive measures in the Netherlands have not changed substantially. Therefore, we could not report on physical activity levels when the restrictions were lifted. This is of particular importance and should be investigated by future studies, as it would give information on whether individuals are able to return to their physical activity levels as they were before the pandemic, or whether it is difficult for people to get back to these levels which might be a call for public health strategies.

## 5. Conclusions

In this population-based study in the Netherlands, we observed low physical activity levels during the COVID-19 pandemic, with most subjects not adhering to physical activity guidelines. For most individuals, the trajectories over a 6-week period after the first restrictive measures were announced remained relatively stable over time during the pandemic, except for a small group of participants that increased their physical activity levels. Determinants of these trajectories included demographics, physical and psychosocial health, and lifestyle factors. More specifically, older, lower educated, and those reporting poorer physical health and lifestyle, and more depressive symptoms were more often in trajectories representing lower levels of physical activity. These groups may therefore require additional preventive interventions to promote physical activity.

## Figures and Tables

**Table 1:** Study sample characteristics.

	Total (N = 5777)
	Median (IQR)/N(%)
Demographics	
Sex (female)	3351 (58.0%)
Age (years)-*mean (SD)*	69.4 (11.5)
Educational level	
Primary	355 (6.1%)
Lower	1904 (33.0%)
Further/intermediate	1860 (32.2%)
Higher	1604 (27.8%)
Occupational status	
Employed	1611 (27.9%)
Retired	3475 (60.2%)
Unemployed	167 (2.9%)
Physical health	
Self-perceived health	
Poor/fair	800 (13.8%)
Good	3285 (56.9%)
Very good/excellent	1598 (27.7%)
Psychosocial health	
Depressive symptoms (CES-D)	4.0 (2.0–8.0)
Anxiety symptoms (HADS)	3.0 (1.0–5.0)
Worries about the pandemic (scale 0–10)	5.0 (3.0–7.0)
Lifestyle	
Diet compared to prior	
(Much) less healthy	265 (4.6%)
As healthy	5031 (87.1%)
(Much) more healthy	449 (7.8%)
Smoking	595 (10.3%)
Alcohol (glasses/day)	1.0 (0.0–2.0)
Physical activity (Moderate-to-vigorous, past 14 days)	
Less than 50 min	1323 (22.9%)
50–100 min	1234 (21.4%)
100–150 min	877 (15.2%)
150–180 min	670 (11.6%)
More than 180 min	1673 (29.0%)

Data are presented as median (IQR) or N(%) unless otherwise indicated, and shown for non-imputed data. Missing data was low (<3%, except for worries about coronacrisis (4.3%) and occupational status (9.1%). IQR = Interquartile Range, SD = Standard Deviation, CES-D = Center for Epidemiologic Studies Depression, HADS = Hospital Anxiety and Depression scale.

**Table 2:** Sex- and age adjusted associations of potential determinants with the latent class of physical activity.

	‘Steadily High’ (N = 1837)	‘Increasers’ (N = 287)	‘Steadily Medium’ (N = 1277)	‘Steadily Low’ (N = 1590)	‘Steadily Very Low’ (N = 786)
	Ref	OR (95% CI)	OR (95% CI)	OR (95% CI)	OR (95% CI)
Demographics					
Sex (ref = male)	ref				
Female		0.95 (0.74–1.22)	1.16 (1.01–1.34) *	1.27 (1.11–1.46) *	1.43 (1.20–1.70) *
Age (per 10 years)	ref	0.97 (0.87–1.08)	1.23 (1.15–1.31) *	1.44 (1.36–1.53) *	1.82 (1.68–1.97) *
Educational level (ref = higher)					
Primary	ref	2.21 (1.29–3.78) *	1.74 (1.21–2.51) *	2.35 (1.68–3.28) *	4.84 (3.33–7.03) *
Lower	ref	1.00 (0.71–1.41)	1.50 (1.24–1.82) *	1.64 (1.36–1.97) *	2.24 (1.76–2.86) *
Further/intermediate	ref	1.12 (0.83–1.52)	1.37 (1.14–1.65) *	1.56 (1.31–1.86) *	1.76 (1.38–2.25) *
Occupational status (ref = employed)					
Unemployed	ref	2.07 (1.12–3.82) *	1.20 (0.77–1.88)	1.61 (1.09–2.39) *	1.76 (1.02–3.03) *
Retired	ref	0.83 (0.53–1.29)	0.76 (0.59–0.99) *	0.59 (0.46–0.75) *	0.47 (0.34–0.64) *
Physical health					
Self-perceived health (ref = very good/excellent)					
Poor/fair	ref	2.42 (1.50–3.90) *	2.09 (1.57–2.78) *	5.00 (3.85–6.49) *	13.17 (9.66–17.95) *
Good	ref	1.70 (1.28–2.26) *	1.39 (1.19–1.63) *	2.08 (1.77–2.44) *	2.50 (1.96–3.18) *
Psychosocial health					
Depressive symptoms	ref	1.05 (1.02–1.08) *	1.05 (1.03–1.07) *	1.08 (1.06–1.09) *	1.13 (1.11–1.15) *
Anxiety symptoms	ref	1.03 (0.99–1.07)	1.02 (1.00–1.05)	1.05 (1.03–1.07) *	1.09 (1.06–1.12) *
Worries about the pandemic (scale 0–10)	ref	1.08 (1.02–1.14) *	1.02 (0.99–1.06)	1.06 (1.–1.09) *	1.03 (0.99–1.06)
Lifestyle					
Diet compared to prior (ref = as healthy)					
(Much) less healthy	ref	1.79 (1.02–3.13) *	1.29 (0.88–1.90)	1.85 (1.30–2.62) *	3.36 (2.29–4.94) *
(Much) more healthy	ref	1.04 (0.66–1.64)	1.20 (0.92–1.55)	1.00 (0.77–1.30)	1.17 (0.84–1.63)
Smoking	ref	1.09 (0.71–1.68)	1.46 (1.14–1.87) *	1.40 (1.11–1.78) *	2.72 (2.08–3.55) *
Alcohol (glass/day)	ref	0.97 (0.88–1.07)	1.00 (0.94–1.05)	0.90 (0.85–0.95) *	0.86 (0.79–0.93) *

OR = Odds Ratio, CI = Confidence Interval, CES-D = Center for Epidemiologic Studies Depression, HADS = Hospital Anxiety and Depression scale, * *p*-value < 0.05.

**Table 3:** Multivariate adjusted associations of potential determinants with the latent class of physical activity.

	‘Steadily High’ (N = 1837)	‘Increasers’ (N = 287)	‘Steadily Medium’ (N = 1277)	‘Steadily Low’ (N = 1590)	‘Steadily Very Low’ (N = 786)
	Ref	OR (95% CI)	OR (95% CI)	OR (95% CI)	OR (95% CI)
Demographics					
Sex (ref = male)					
Female	ref	0.87 (0.66–1.13)	1.08 (0.93–1.26)	1.10 (0.94–1.28)	1.18 (0.97–1.43)
Age (per 10 years)	ref	1.04 (0.86–1.27)	1.34 (1.20–1.51) *	1.69 (1.51–1.88) *	2.22 (1.94–2.54) *
Educational level (ref = higher)					
Primary	ref	1.94 (1.13–3.35) *	1.61 (1.11–2.33) *	2.01 (1.43–2.82) *	3.64 (2.45–5.40) *
Lower	ref	0.94 (0.67–1.32)	1.45 (1.20–1.76) *	1.51 (1.25–1.82) *	1.95 (1.51–2.51) *
Further/intermediate	ref	1.05 (0.77–1.43)	1.31 (1.09–1.57) *	1.39 (1.16–1.66) *	1.43 (1.11–1.85) *
Occupational status (ref = employed)					
Unemployed	ref	1.73 (0.92–3.26)	0.93 (0.59–1.47)	1.11 (0.74–1.68)	0.84 (0.47–1.51)
Retired	ref	0.79 (0.50–1.23)	0.72 (0.55–0.94) *	0.52 (0.41–0.68) *	0.38 (0.27–0.53) *
Physical health					
Self-perceived health (ref = very good/excellent)					
Poor/fair	ref	1.96 (1.17–3.30) *	1.70 (1.24–2.31) *	3.89 (2.93–5.17) *	8.56 (6.08–12.08) *
Good	ref	1.56 (1.16–2.09) *	1.29 (1.10–1.52) *	1.88 (1.59–2.22) *	2.16 (1.68–2.78) *
Psychosocial health					
Depressive symptoms	ref	1.06 (1.01–1.11) *	1.07 (1.04–1.10) *	1.07 (1.05–1.10) *	1.13 (1.09–1.17) *
Anxiety symptoms	ref	0.93 (0.87–0.99) *	0.94 (0.90–0.97) *	0.93 (0.89–0.96) *	0.90 (0.86–0.94) *
Worries about the pandemic (scale 0–10)	ref	1.07 (1.01–1.14) *	1.01 (0.97–1.04)	1.03 (1.00–1.07)	0.96 (0.92–1.00)
Lifestyle					
Diet compared to prior (ref = as healthy)					
(Much) less healthy	ref	1.50 (0.85–2.64)	1.12 (0.76–1.67)	1.43 (0.99–2.05)	2.17 (1.44–3.27) *
(Much) more healthy	ref	1.00 (0.64–1.59)	1.17 (0.90–1.52)	0.94 (0.72–1.23)	1.00 (0.71–1.43)
Smoking	ref	1.00 (0.65–1.56)	1.36 (1.06–1.74) *	1.27 (0.99–1.63)	2.30 (1.73–3.05) *
Alcohol (glass/day)	ref	0.97 (0.88–1.07)	1.00 (0.94–1.06)	0.91 (0.86–0.96) *	0.89 (0.82–0.96) *

OR = Odds Ratio, CI = Confidence Interval, CES-D = Center for Epidemiologic Studies Depression, HADS = Hospital Anxiety and Depression scale, * *p*-value < 0.05.

## Data Availability

Data can be obtained upon request. Requests should be directed towards the management team of the Rotterdam Study (secretariat.epi@erasmusmc.nl), which has a protocol for approving data requests. Because of restrictions based on privacy regulations and informed consent of the participants, data cannot be made freely available in a public repository.

## Supplementary Materials

The following are available online at https://www.mdpi.com/article/10.3390/nu13113832/s1, Table S1: Descriptive information on physical activity of each time point, Table S2: Study sample characteristics per trajectory of physical activity, Table S3: Multivariate adjusted associations of potential determinants for the ‘increasers’ trajectory.
