# Supplementary material for: Trajectories and Determinants of Physical Activity during COVID-19 Pandemic: A Population-Based Study of Middle-Aged and Elderly Individuals in The Netherlands
Source: Nutrients. 2021 Oct 27;13(11):3832. doi: 10.3390/nu13113832 (PMC8618734; doi:10.3390/nu13113832)
Supplement: Supplementary file 1 [file nutrients-13-03832-s001.zip › nutrients-1429019-SI.pdf]

SUPPLEMENTARY MATERIAL

Trajectories and Determinants of Physical Activity during Covid-19 Pandemic: A Population-Based Study of Middle-Aged and Elderly Individuals in The Netherlands

Amy Hofman[1], Marlou A.M. Limpens[1#], Tosca O.E. Crom[1#], M. Arfan Ikram[1], Annemarie I. Luik[1,2], Trudy Voortman[1,3]

[#] Marlou A.M. Limpens and Tosca O.E. de Crom contributed equally

[1] Department of Epidemiology, Erasmus MC, University Medical Center Rotterdam, Rotterdam, The Netherlands,

[2] Department of Child and Adolescent Psychiatry/Psychology, Erasmus MC University Medical Center Rotterdam, Rotterdam, the Netherlands,

[3] Division of Human Nutrition and Health, Wageningen University & Research, Wageningen, The Netherlands

Supplementary material

Supplemental Table S1. Descriptive information on physical activity of each time point.

	**(Moderate-to-Vigorous) Physical Activity during the Past 14 Days**		
	**Time Point 1 N(%)**	**Time Point 2 N(%)**	**Time Point 3 N(%)**
Less than 50 minutes	1323 (22.9%)	689 (14.2%)	545 (11.8%)
50–100 minutes	1234 (21.4%)	798 (16.5%)	741 (16.0%)
100–150 minutes	877 (15.2%)	614 (12.7%)	593 (12.8%)
150–180 minutes	670 (11.6%)	547 (11.3%)	533 (11.5%)
More than 180 minutes	1673 (29.0%)	2190 (45.3%)	2211 (47.8%)
Total	5777 (100.0%)	4838 (100.0%)	4623 (100.0%)

Question asked at each time point: "During the past 14 days, how many minutes did you exercise (moderately) intensively in total (walking, cycling, running)?"

Supplemental Table S2. Study sample characteristics per latent class trajectory of physical activity.

	'Steadily High' (N = 1837)	'Increasers' (N = 287)	'Steadily Medium' (N = 1277)	'Steadily Low' (N = 1590)	'Steadily very Low' (N = 786)
	**Median (IQR)/N(%)**	**Median (IQR)/N(%)**	**Median (IQR)/N(%)**	**Median (IQR)/N(%)**	**Median (IQR)/N(%)**
**Demographics**					
Sex (female)	1006 (54.8%)	154 (53.7%)	743 (58.2%)	956 (60.1%)	492 (62.6%)
Age *mean(SD)*	66.6 (10.1)	66.2 (10.5)	69.2 (11.3)	71.2 (11.8)	73.9 (12.2)
Educational level					
Primary	68 (3.7%)	21 (7.3%)	67 (5.2%)	108 (6.8%)	91 (11.6%)
Lower	503 (27.4%)	71 (24.7%)	435 (34.1%)	570 (35.8%)	325 (41.3%)
Further/intermediate	587 (32.0%)	94 (32.8%)	421 (33.0%)	529 (33.3%)	229 (29.1%)
Higher	668 (36.4%)	97 (33.8%)	339 (26.5%)	366 (23.0%)	134 (17.0%)
Occupational status					
Employed	645 (35.1%)	101 (35.2%)	357 (28.0%)	366 (23.0%)	142 (18.1%)
Retired	1015 (55.3%)	147 (51.2%)	780 (61.1%)	1012 (63.6%)	521 (66.3%)
Unemployed	47 (2.6%)	15 (5.2%)	34 (2.7%)	49 (3.1%)	22 (2.8%)
**Physical health**					
Self-perceived health					
Poor/fair	110 (6.0%)	28 (9.8%)	129 (10.1%)	273 (17.2%)	260 (33.1%)
Good	980 (53.3%)	177 (61.7%)	753 (59.0%)	970 (61.0%)	405 (51.5%)
Very good/excellent	726 (39.5%)	79 (27.5%)	378 (29.6%)	312 (19.6%)	103 (13.1%)
**Psychosocial health**					
Depressive symptoms (CES-D)	3.00 (1.00–6.00)	4.00 (1.00–8.00)	4.00 (2.00–8.00)	5.00 (2.00–8.00)	6.00 (3.00–10.00)
Anxiety symptoms (HADS)	2.00 (1.00–5.00)	2.00 (1.00–5.00)	3.00 (1.00–5.00)	3.00 (1.00–5.00)	4.00 (1.00–6.00)
Worries about the pandemic (scale 0–10)	5.00 (3.00–7.00)	5.00 (3.00–7.00)	5.00 (3.00–7.00)	5.00 (3.00–7.00)	5.00 (3.00–7.00)
**Lifestyle**					
Diet compared to prior					
(Much) less healthy	62 (3.4%)	17 (5.9%)	48 (3.8%)	78 (4.9%)	60 (7.6%)
As healthy	1619 (88.1%)	246 (85.7%)	1110 (86.9%)	1390 (87.4%)	666 (84.7%)
(Much) more healthy	150 (8.2%)	24 (8.4%)	112 (8.8%)	109 (6.9%)	54 (6.9%)
Smoking	158 (8.6%)	27 (9.4%)	138 (10.8%)	154 (9.7%)	118 (15.0%)
Alcohol (glasses/day)	1.00 (0.00–2.00)	1.00 (0.00–2.00)	1.00 (0.00–2.00)	1.00 (0.00–1.00)	0.00 (0.00–1.00)
Physical activity (Moderate-to-vigorous, past 14 days)					
Less than 50 minutes	0 (0.0%)	94 (32.8%)	0 (0.0%)	479 (30.1%)	750 (95.4%)
50–100 minutes	0 (0.0%)	193 (67.2%)	143 (11.2%)	862 (54.2%)	36 (4.6%)
100–150 minutes	179 (9.7%)	0 (0.0%)	508 (39.8%)	190 (11.9%)	0 (0.0%)
150–180 minutes	305 (16.6%)	0 (0.0%)	316 (24.7%)	49 (3.1%)	0 (0.0%)
More than 180 minutes	1353 (73.7%)	0 (0.0%)	310 (24.3%)	10 (0.6%)	0 (0.0%)

Data are presented as median(IQR) or N(%) unless otherwise indicated, and shown for non-imputed data. Missing data was low (<3%, except for worries about coronacrisis (4.3%) and occupational status (9.1%). IQR = Interquartile Range, SD = Standard Deviation, , CES-D = Center for Epidemiologic Studies Depression, HADS = Hospital Anxiety and Depression scale.

Supplemental Table S3. Multivariate adjusted associations of potential determinants for the 'increasers' trajectory.

	'Steadily Low' (N = 1590)	'Increasers' (N = 287)
		OR (95%CI)
**Demographics**		
Sex (ref = male)		
Female	ref	0.79 (0.60–1.03)
Age (per 10 years)	ref	0.62 (0.51–0.75) *
Educational level (ref = higher)		
Primary	ref	0.97 (0.57–1.65)
Lower	ref	0.62 (0.44–0.88) *
Further/intermediate	ref	0.76 (0.55–1.04)
Occupational status (ref = employed)		
Unemployed	ref	1.56 (0.84–2.91)
Retired	ref	1.50 (0.96–2.36)
**Physical health**		
Self-perceived health (ref = very		
good/excellent)	ref	0.50 (0.30–0.84) *
Poor/fair	ref	0.83 (0.61–1.13)
Good		
**Psychosocial health**		
Depressive symptoms	ref	0.98 (0.94–1.03)
Anxiety symptoms	ref	1.00 (0.94–1.07)
Worries about the pandemic (scale 0–10)	ref	1.04 (0.97–1.10)
**Lifestyle**		
Diet compared to prior (ref = as healthy)		
(Much) less healthy	ref	1.05 (0.60–1.83)
(Much) more healthy	ref	1.07 (0.67–1.72)
Smoking	ref	0.79 (0.51–1.23)
Alcohol (glass/day)	ref	1.07 (0.97–1.19)

OR = Odds Ratio, CI = Confidence Interval, ref = reference, CES-D = Center for Epidemiologic Studies Depression, HADS = Hospital Anxiety and Depression scale.
